# Multiscale Analysis of Size Effect of Surface Pit Defect in Nanoindentation

**DOI:** 10.3390/mi9060298

**Published:** 2018-06-13

**Authors:** Zhongli Zhang, Yushan Ni, Jinming Zhang, Can Wang, Xuedi Ren

**Affiliations:** 1 Department of Aeronautics and Astronautics, Fudan University, Shanghai 200433, China; 17110290016@fudan.edu.cn; 2 Institute of Measurement and Testing Technology, Shanghai 201203, China; zhangjm@simt.com.cn (J.Z.); wangc@simt.com.cn (C.W.); renxd@simt.com.cn (X.R.)

**Keywords:** multiscale, quasicontinuum method, surface pit defect, size effect

## Abstract

The nanoindentation on a pit surface has been simulated using the quasicontinuum method in order to investigate the size effect of surface pit defect on the yield load of thin film. Various widths and heights of surface pit defect have been taken into account. The size coefficient has been defined as an index to express the influence of the width or height of surface pit defect. The results show that as the size coefficient of width (of height) increases, at first the yield load of thin film decreases extremely slowly, until the size coefficient of width equals approximately one unit (half unit), at which point the yield load experiences an obvious drop. When the size coefficient of width (of height) reaches approximately two units (one unit), the yield load is almost the same as that of the nanoindentation on a stepped surface. In addition, the height of surface pit defect has more influence than the width on the yield load of thin film.

## 1. Introduction

As the development of a nanotechnique, nanoindentation [1] has already been a relatively simple and effective method for evaluating the material property of thin films. In order to get closer to the real system, a number of scientists have recently studied nanoindentation through simulations and experiments on thin film with defects such as inhomogeneities [2], grain boundaries [3,4], surface scratches [5], and surface steps [6,7]. Victor V. Pogorelko et al. [8] have found that nanohardness of coating is less than that of a single crystal Cu due to defects through their simulation. Telmo G. Santos et al. [9] have investigated how to identify micro- and nano-surface defects. Additionally, various kinds of surface defects have been artificially made to probe to friction and surface roughness phenomenon [10,11,12]. It is known to all that surface pit defect is very common in epitaxial thin films [13,14,15] and nanoimprint technology [16,17,18]. In fact, surface roughness has also been treated as part of the mixed group of surface pit defects [19]. Therefore, some scientific workers have focused on a pit surface to gain more information for the actual application of materials. Ni yushan et al. [20] studied nanoindentation of Al thin film compared with a surface defect situation and a defect free situation. The result shows that the initial surface defect has an obvious delay effect for the second dislocation emission, which indicates that a pit surface indeed plays an important role in the property of materials. Thus, it is necessary and significant to probe the influence of a pit surface in nanoindentation.

As the computer technology is highly developed, simulation methods, such as molecular dynamics (MD), become an efficient way to simulate nanoindentation experiments. However, the calculation accuracy and simulation efficiency of MD are limited by computer hardware, which make it impossible to use for large scale simulation. The quasicontinuum method (QC) is a multiscale method that combines continuum mechanics and molecular mechanics. It applies the MD model at the intense deformation region and a finite element model elsewhere so that the efficiency of the simulation is greatly improved to ensure accuracy.

It is difficult to study the nanoindentation on a pit surface through experiments. Thus far, relevant studies on this aspect are still rare to find. Our aim is to investigate the size effect of surface pit defect on the yield load in nanoindentation using the QC method, and hope it is an important directive to the defects testing or material application.

## 2. Methodology

The quasicontinuum method is applied in this simulation, which is brought by Tadmor [21]. It is one of the multiscale approaches that it keeps an atomistic description at highly deformed regions, whereas a linear elastic continuum method is implemented far away from this dislocation core. The QC method proceeds through molecular static energy minimization over an atomistic (non-local) domain and a finite element (local) domain. In this simulation, the Ercolessi–Adams potential (EAM) [22] is employed to describe the atomistic behavior of the system, and a finite element method is used at the linear deformation area of the material.

**Figure 1:**
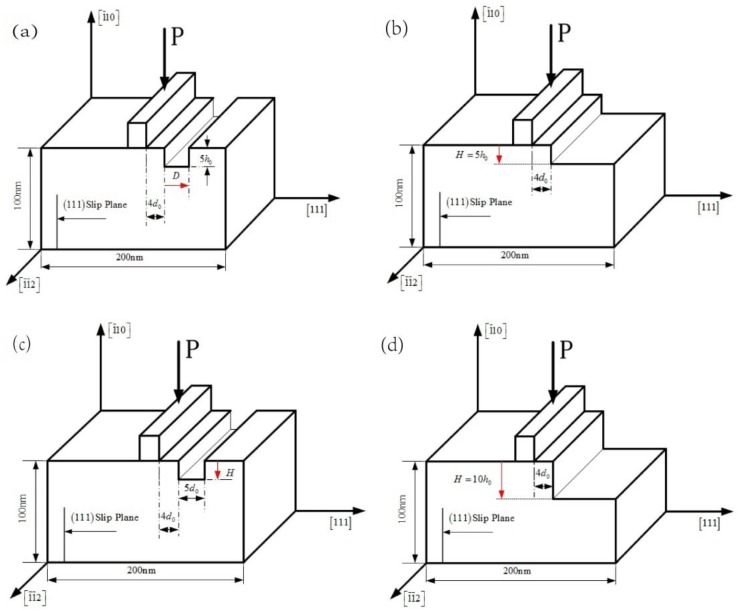
Schematic representation of the nanoindentation model of size effect: (**a**) width (D) changing from 1d_0_ to 10d_0_ of surface pit defect with the fixed height = 5h_0_; (**b**) the comparison model of surface step with height = 5h_0_; (**c**) height (H) changing from 1h_0_ to 10h_0_ of surface pit defect with the fixed width = 5d_0_; (**d**) the comparison model of surface step with height = 10h_0_.

Figure 1 shows the nanoindentation model used in the simulation. The *x*-axis direction is [1 1 1] direction, the *y*-axis direction is [$\bar{1}$ 1 0], and the outer-of-plane *z* direction is [$\bar{1}$ $\bar{1}$ 2] direction. This orientation is selected to facilitate dislocation emission. The width of Al thin film is 200 nm, and the height is 100 nm, which has one order of magnitude bigger than the usual MD level. The indenter is rigid with a width 4d_0_, where d_0_ is 0.2328 nm (one atomic lattice spacing in [1 1 1] direction). The distance between the adjacent boundary of the indenter and surface pit defect is selected to be 4d_0_ (Figure 1), which is proved to be reasonable. When the surface pit defect is too close to the indenter, the deformation of surface pit defect is so severe that the crack is propagated under the tip of surface pit defect; when it is too far away from the indenter, the influence of pit defect on the yield load is too weak. According to the research on nanoindentation on a stepped surface [7], the spatial extent of the step’s influence has been found out to be approximately three times the contact radius (measured mean stress at yield as a function of *d*/*a*_y_; absolute value of distance from the step normalized by elastic contact radius prior to yield) on the surface, having step heights ranging from 5 to 30 Å. When *d*/*a*_y_ < 2, the yield stress is reduced by a neighboring step, while for *d*/*a*_y_ > 3, the yield stress is unaffected. The nanohardness are both calculated by the actual contact radius, though the indentation tip shape in this simulation and the nanoindentation experiment is square and round-like, respectively. Consequently, the change law of the nanohardness is reasonably similar if the ratio of surface defect distance to the actual contact radius is taken into account (just as the experiment discussed). In this simulation, the contact radius is 2d_0_ (half of the indenter width) and the distance between the left boundary of surface pit defect and the centre of the indenter is 6d_0_ (4d_0_ + 4d_0_/2 = 6d_0_), which greatly agrees with this reference (6d_0_/2d_0_=3). Figure 1a,b are the simulation model of the width effect of surface pit defect. Ten different widths in this simulation are shown as D in Figure 1a, namely, 1d_0_, 2d_0_, 3d_0_, 4d_0_, 5d_0_, 6d_0_, 7d_0_, 8d_0_, 9d_0_, and 10d_0_, and the height of surface pit defect is a fixed height 5h_0_; Figure 1b shows the comparison model of surface step with height = 5h_0_; Figure 1c,d are the simulation model of the height effect of surface pit defect, where Figure 1c shows ten different heights H of surface pit defect, namely, 1h_0_, 2h_0_, 3h_0_, 4h_0_, 5h_0_, 6h_0_, 7h_0_, 8h_0_, 9h_0_, and 10h_0_, and the width of surface pit defect is a fixed value 5d_0_; Figure 1(d) shows the comparison model of surface step with height = 10h_0_. These parameters of width and height are selected in order to make a more comprehensive investigation. Further, in the out-of-plane direction, the thickness of this model is equal to the minimal repeat distance with the periodic boundary condition applied. The boundary condition of this single crystal Al thin film keeps it rigid at the bottom and free at the sides. The atoms under the indenter are forced to move gradually into the material by the displacement-imposed boundary condition. Each load step of the indenter is 0.02 nm, with a final depth 1.2 nm, because it is relatively proper and effective to catch the dislocation nucleation and mission with minimum total load steps. Because the width of the indenter is 1/200 of film width and the final depth is 1/100 of film height, it ensures that far-field boundary conditions do not affect the behavior in the vicinity of the indenter.

**Figure 2:**
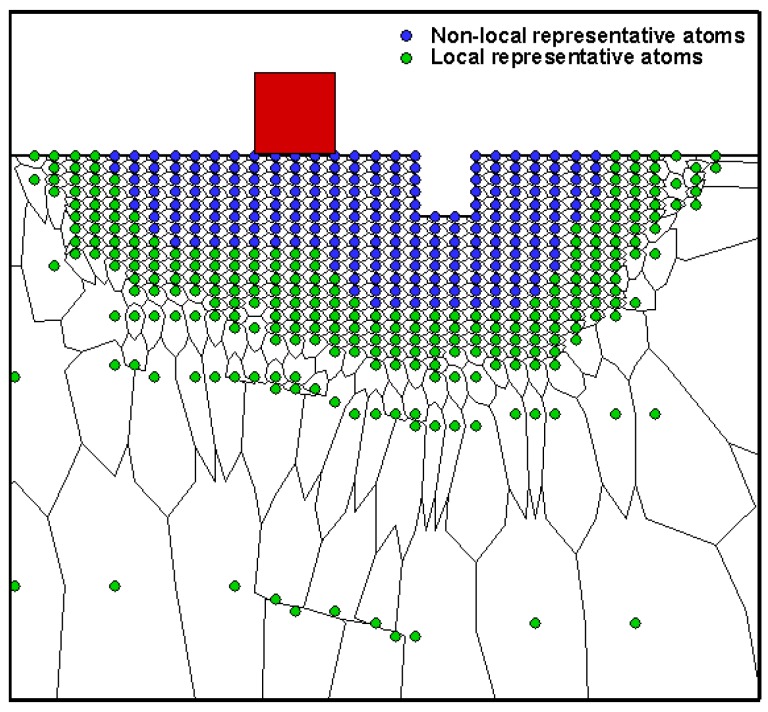
Schematic of local and non-local representative atoms with initial surface pit defect.

The material of the model is single crystal Al thin film, and the crystallographic lattice constant *a*_1_ is 0.4032 nm. One atomic spacing in [$\bar{1}$ 1 0] direction (h_0_) is 0.1426 nm. Burgers vector $\vec{b}$ is 0.285 nm, shear modulus *μ* is 33.14 GPa, Poisson *ν* is 0.319, and (1 1 1) surface energy *γ*_111_ is 0.869 J/m^2^. Figure 2 shows the schematic of local and non-local representative atoms and tessellation during nanoindentation on ($\bar{1}$ $\bar{1}$ 2) plane of Al film with initial surface pit defect, where the red square is the rigid indenter and the blue filled circles are the non-local representative atoms, while the green ones are the local representative atoms. The system investigated here is very large by current atomistic modeling standards. A standard lattice statics analysis for this system would treat millions of atoms and would have to be performed on a parallel supercomputer. By using the quasicontinuum method, the computational intensity is greatly reduced. Regarding this single crystal Al system with a size of 100 nm × 200 nm, only 5000 atoms are treated explicitly at most (15,000 degrees of freedom), and a simulation can be finished on a common personal computer in a few days.

## 3. Results

### 3.1. Width Effect of Surface Pit Defect on Yield Load

**Figure 3:**
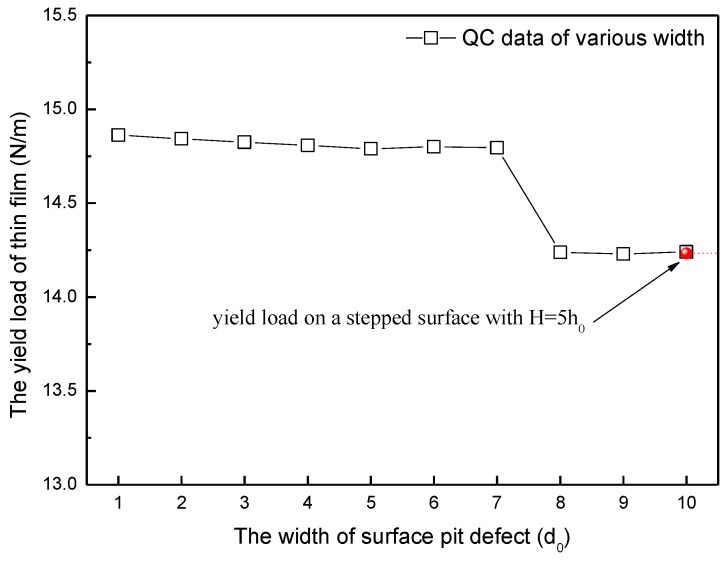
The yield load of thin film as the width changing of surface pit defect (with a standard deviation of 0.01 N/m). QC—quasicontinuum method.

It has long been recognized that the yield load of materials is one of the most important indexes of material properties. At the load-displacement curve, the yield load corresponds to the first highest point at the initial linear portion, which indicates onset of the dislocation emission. Further, the yield load of materials can be obviously influenced by defects such as surface pit defect. In the present paper, ten different widths of surface pit defect are simulated, from D = 1d_0_ to 10d_0_, in order to probe the width effect of surface pit defect on yield load. Figure 3 shows the yield load curve as the width of surface pit defect changes. It can be found out that the yield load of thin film with surface pit defect generally displays a tendency to decrease, which is reasonable because the structure of thin film is destroyed more and more severely by the increase of the width of surface pit defect. When the simulated width increases from D = 1d_0_ to 7d_0_, the yield load decreases extremely slowly; after the width reaches 7d_0_, the yield load experiences an obvious drop from 14.8 N/m to 14.24 N/m. Then, the yield load curve displays the phenomenon of a slow decrease again.

In order to conduct a comprehensive investigation of the width effect of surface pit defect, the nanoindentation on a stepped surface has been carried out for comparison (namely, the simulation width of surface pit defect is infinitely large), as shown in Figure 1b. The results show that the yield load of nanoindentation on a stepped surface with H = 5h_0_ is approximately 14.23 N/m, which is very close to the yield load value of D = 10d_0_ (the red point in Figure 3). That is to say, when the width of surface pit defect increases to 10d_0_, the yield load of thin film almost reaches the yield load value of nanoindentation on a stepped surface.

### 3.2. Height Effect of Surface Pit Defect on Yield Load

**Figure 4:**
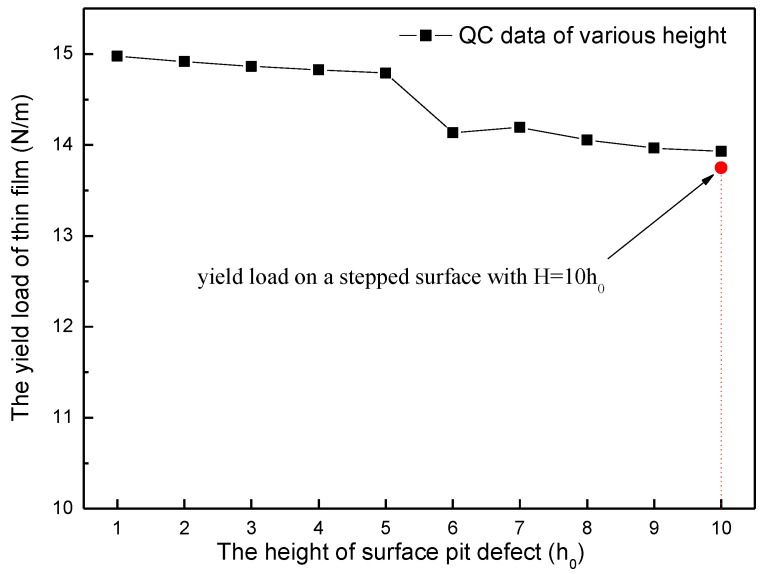
The yield load of thin film as the height changing of surface pit defect (with a standard deviation of 0.01 N/m).

An investigation of the height effect of surface pit defect on yield load has also been carried out. Ten different heights of surface pit defect are simulated, from H = 1h_0_ to 10h_0_, with a fixed width D = 5d_0_ (as shown in Figure 1c). Figure 4 shows the yield load curve as the height of surface pit defect changes. It can be found out that the change law of the yield load of thin film is very similar to the situation of the width effect. As the simulation height increases from H = 1h_0_ to 5h_0_, the yield load decreases extremely slowly, until the height reaches 6h_0_, at which point the yield load experiences an obvious drop from 14.79 N/m to 14.14 N/m. Then, the yield load curve slowly decreases again.

The nanoindentation on a stepped surface with the 10h_0_ step height has been investigated for comparison, as shown in Figure 1d. The results show that the yield load of nanoindentation on such a stepped surface is approximately 13.75 N/m (the red point in Figure 4). It can be easily found out that when the simulation height of surface pit defect increases to 10h_0_, the yield load of thin film is about 13.93 N/m, which is already close to the yield load of nanoindentation on a stepped surface.

## 4. Discussion

### 4.1. The Investigation of Dislocation Nucleation and the Estimation of Peierls Stress

**Figure 5:**
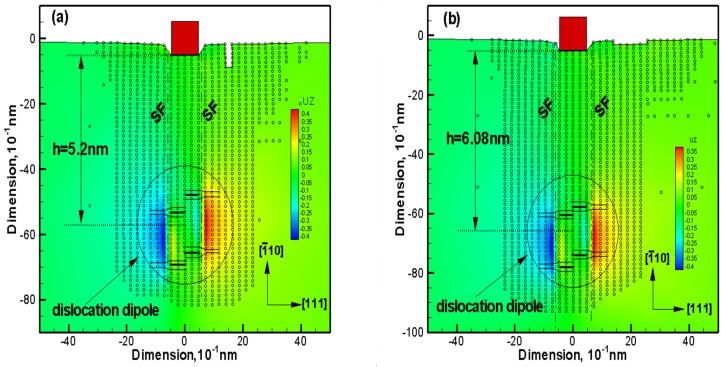
Snapshot of atoms under the indenter and corresponding out-of-plane displacement plot, where UZ is atom displacement at out-of-plane: (**a**) width changing D = 1d_0_ at the yield of thin film; (**b**) height changing H = 1h_0_ at the yield of thin film.

In order to probe the reason for such an obvious decline of yield load (D = 7d_0_ to 8d_0_ section in Figure 3, H = 5h_0_ to 6h_0_ section in Figure 4), relevant snapshot of atoms under the indenter and corresponding out-of-plane displacement plot are probed. The results show that when the thin film yields, two dissociated <1 1 0> edge dislocations are emitted beneath the indenter after nucleation. Considering there are too many snapshots, the situation of D = 1d_0_ in width effect simulation and H = 1h_0_ in height effect simulation are carried out for example. The dislocated structure beneath the indenter is given in Figure 5, along with the out-of-plane displacements experienced by the atoms, where dimensions and displacements are in 0.1 nm. The nucleated dislocations are easily seen through UZ contours displayed in Figure 5. The out-of-plane displacements in the stacking fault regions between the partials are a clear fingerprint of the location of the dislocations. The repeat distance in the out-of-plane direction of the crystal structure is 0.4938 nm for this model. It can be found out that the dislocations are composed of 1/6 <1 1 2> Shockley partials that bound a stacking fault. On the left,

(1) $$\frac{1}{2}[\bar{1}10]=\underset{\mathrm{top}}{\underset{︸}{\frac{1}{6}[\bar{1}2\bar{1}]}}+\underset{\mathrm{bottom}}{\underset{︸}{\frac{1}{6}[\bar{2}11]}}$$

and on the right,

(2) $$\frac{1}{2}[1\bar{1}0]=\underset{\mathrm{top}}{\underset{︸}{\frac{1}{6}[1\bar{2}1]}}+\underset{\mathrm{bottom}}{\underset{︸}{\frac{1}{6}[2\bar{1}\bar{1}]}}$$

In Figure 5a, the dislocation dipole travels into bulk after nucleation at the load step of 0.5 nm, and its centre settles at the depth of 5.2 nm. In Figure 5b, the dislocation dipole travels into bulk at the same load step of 0.5 nm, but its centre settles at the depth of 6.08 nm. Further, when compared with all these snapshots of atom structures in the simulation of size effect, it is found out that when the size of surface pit defect changes, there is a different emission depth of dislocations (see Figure 5a,b, for example). That is to say, most likely the different yield load of thin film in macroscopy corresponds to the emission depth of dislocation in microscopy.

For the purpose of the explanation, such change law of the yield load, these emission depths of dislocations are used as an equilibrium distance to further obtain an estimate for the Peierls stress predicted by the EAM potential [22]. Because Peierls stress is actually the resisting force during the dislocation movement resulting from the lattice structure, the change of the yield load can be reasonably explained by Peierls stress. Aside from the lattice friction, there are two forces acting on the dislocation: (i) the Peach–Koehler force (*F*_PK_) due to the indenter stress field driving the dislocation into bulk; (ii) the image force (*F*_I_) pulling the dislocation to the surface. The force on the dislocation is the sum of these two forces. The dislocation escapes the attractive region and propagates into the bulk, and is finally stopped by lattice friction. That is to say, the force on the dislocation will be balanced at the equilibrium depth by the lattice friction force that results from the Peierls stress ($\sigma_{p}$) [23].

(3) $$F_{\mathrm{PK}}+F_{Ι}=b\sigma_{p}$$

**Figure 6:**
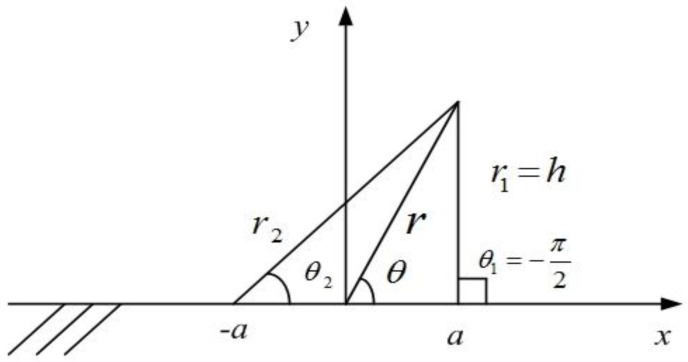
Bipolar coordinate for a 2*a* indentation contact.

To compute the Peach–Koehler force, shear stress field is required beneath the indenter. In this simulation, there is a frictionless rectangular indenter acting on an elastic body occupying the lower half-plane, *y* < 0, the shear stress in bipolar coordinates is [24]

(4) $$\sigma_{\mathrm{xy}}=-\frac{Pr^{2}\sin\theta }{\pi {\left(r_{1}r_{2}\right)}^{3/2}}\sin[\theta -\frac{3}{2}(\theta_{1}+\theta_{2})]$$

where *P* is the indentation load. According to the coordinate system of 2*a* indentation contact (the width of indenter is 2*a*), as shown in Figure 6, at a depth *h* beneath the right indenter tip, there is $r=\sqrt{a^{2}+h^{2}}$, $r_{1}=h$, $r_{2}=\sqrt{4a^{2}+h^{2}}$, $\theta =-\tan^{-1}h/a$, $\theta_{1}=-\pi /2$, $\theta_{2}=-\tan^{-1}(h/2a)$. The resulting Peach–Koehler force is

(5) $$F_{\mathrm{PK}}(h)=(b\cdot \sigma )\times \ell =b\sigma_{\mathrm{xy}}(h)$$

where ***b*** is the Burgers vector, σ is the applied stress tensor, and ℓ is the dislocation line vector.

The image force acting on one of the dislocations of a dipole of width *d* = 2*a* at depth *h* beneath the indenter can be shown to be

(6) $$F_{Ι}=\frac{\mu b^{2}}{\pi (1-v)}[\frac{1}{4h}-\frac{4h^{3}(4h^{2}-3d^{2})}{{\left(4h^{2}+d^{2}\right)}^{3}}]$$

**Figure 7:**
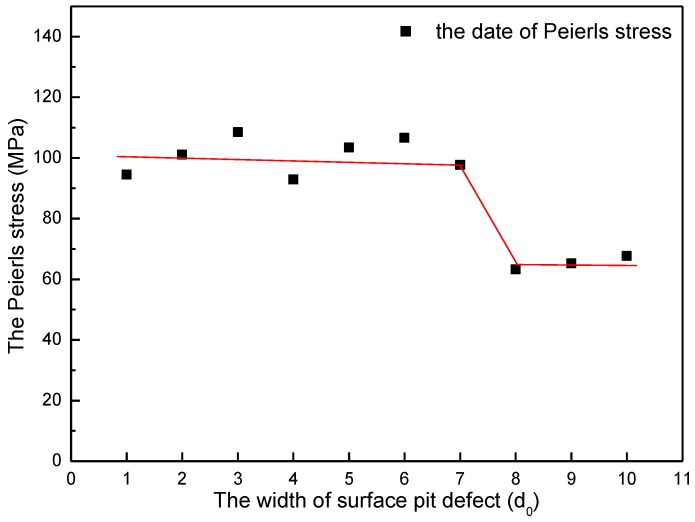
The variation of Peierls stress in the simulation of width effect (with a standard deviation of 0.2 MPa).

**Figure 8:**
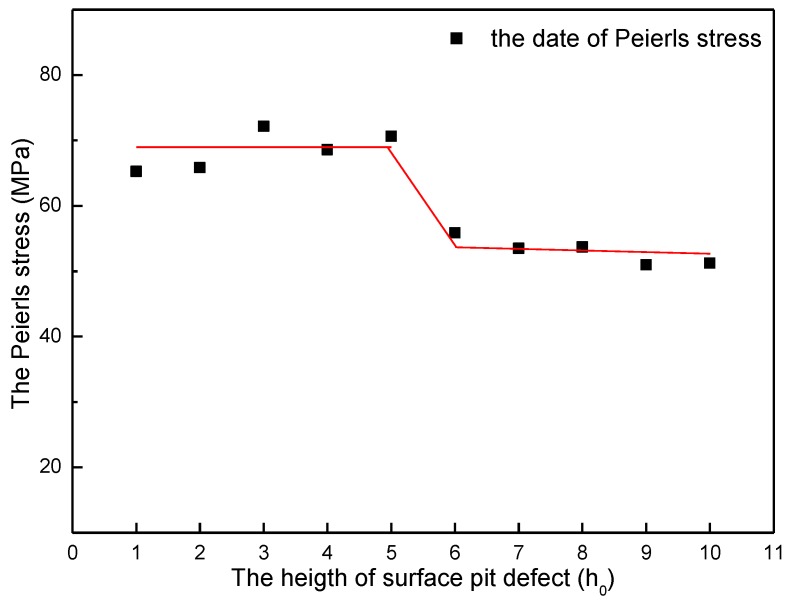
The variation of Peierls stress in the simulation of height effect (with a standard deviation of 0.1 MPa).

According to the discussion above, Peierls stress in every size of surface pit defect has been calculated and plotted. Figure 7 shows the variation of Peierls stress in the simulation of width effect. It can be easily found out that when the width of surface pit defect changes from D = 1d_0_ to 7d_0_, the Peierls stress fluctuates narrowly at the value of 100 MPa. When the width increases to more than 8d_0_, the Peierls stress abruptly obviously drops down to about 70 MPa. Such change law is greatly in keeping with the variation of yield load in the width effect simulation. In a similar manner, it can be also found out from Figure 8 that when the height of surface pit defect changes from H = 1h_0_ to 5h_0_, the Peierls stress fluctuates narrowly at the value of 70 MPa. When the height increases to more than 6h_0_, the Peierls stress abruptly obviously drops down to about 50 MPa, which is also in accordance with the variation of yield load in the height effect simulation. That is to say, such an obvious decline of yield load (D = 7d_0_ to 8d_0_ section in Figure 3, H = 5h_0_ to 6h_0_ section in Figure 4) results from the severe reduction of the Peierls stress, which is caused by the size increase of surface pit defect. Consequently, it is reasonable and useful to explain the variation of yield load through the Peierls stress.

### 4.2. Size Coefficient

It can be figured out that the turning point (D = 7d_0_ in the width effect simulation while H = 5h_0_ in the height effect simulation) is different in this simulation. That is to say, the influence degree of width parameter is different from the height parameter of surface pit defect. Thus, a further discussion is carried out to quantify the size effect of surface pit defect. It is reasonable that the influence on the hardness and yield load of thin film would be much more severe if the surface pit defect gets closer to the indenter. That is to say, if the same degree of hardness damage is made by surface pit defect, the larger size of pit defect is needed where it is farther away from the indenter. Consequently, in order to define a more precise expression of the size effect of surface pit defect, a size coefficient *α* should be carried out as follows:

(7) $$\alpha =\frac{L^{*}}{d^{*}}$$

where “L*” means the characteristic length of surface pit defect (namely the width D in the width effect simulation and the height H in the height effect simulation), and “d*” means the distance between the center of the indenter and the left boundary of the surface pit defect (in this simulation, d* is a constant 6d_0_).

In the width effect simulation, the critical width of an abrupt obvious drop of yield load is 7d_0_ (at the point D = 7d_0_ in Figure 3). Thus, the size coefficient *α* is approximately 1.17 ($\frac{L^{*}}{d^{*}}=\frac{D}{d^{*}}=\frac{7d_{0}}{6d_{0}}=\frac{7}{6}$). When *α* reaches approximately 2 ($\frac{L^{*}}{d^{*}}=\frac{D}{d^{*}}=\frac{10d_{0}}{6d_{0}}=1.7$), as shown in Figure 3 at the point D = 10d_0_, the yield load of thin film is almost the same with that of nanoindentation on a stepped surface (the red point in Figure 3).

In the height effect simulation, the critical height of an abrupt obvious drop of yield load is 5h_0_ (at the point H = 5h_0_ in Figure 4). Then, the size coefficient *α* is approximately 0.51 ($\frac{L^{*}}{d^{*}}=\frac{H}{d^{*}}=\frac{5h_{0}}{6d_{0}}=0.51$). When *α* reaches approximately 1 ($\frac{L^{*}}{d^{*}}=\frac{H}{d^{*}}=\frac{10h_{0}}{6d_{0}}=1.02$), as shown in Figure 4 at the point H = 10h_0_, the yield load of thin film is almost the same as that of nanoindentation on a stepped surface (the red point in Figure 4).

It can be found out that the size coefficient of height is almost half of the size coefficient of width in the abrupt obvious drop point of yield load decline, which suggests that the height parameter of surface pit defect plays a more important role than width parameter.

**Figure 9:**
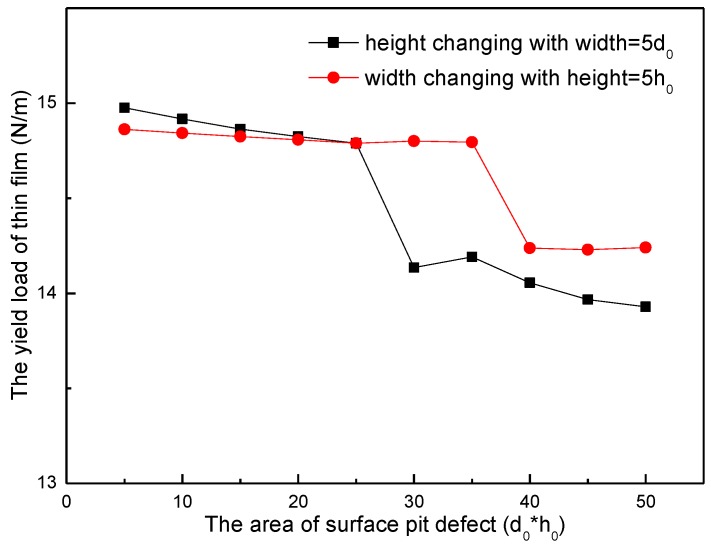
The yield load of thin film as the area changing of surface pit defect.

In addition, from the point of the area of surface pit defect, it also can be proved that the height of surface pit defect is a leading factor on yield load. Figure 9 shows the yield load of thin film changing as the area changes. It can be easily found out that the slope of yield load curve through the increase of height is bigger than the one through the increase of width. It indicates that the increase of height makes the yield load decrease faster. When the area of surface pit defect increases from 5h_0_d_0_ to 25h_0_d_0_, the yield load through the increase of width is smaller than the one through the increase of height. This is because during this internal area, the height value of surface pit defect in the curve of height increase (red curve in Figure 9) is bigger than the other one (black curve). However, when the area is larger than 25h_0_d_0_, the yield load through the increase of height is smaller than the one through the increase of width. This is because the height of surface pit defect in the curve of height increase is over 6h_0_, while the height of surface pit defect in the curve of width increase is still 5h_0_. According to the discussion above, the height of surface pit defect makes more influence than width in the yield load of thin film, which indicates that the height of the pit is a leading factor on the influence of the yield load in nanoindentation.

## 5. Conclusions

In this paper, the QC method is employed to investigate the size effect of surface pit defect on yield load in nanoindentation. The conclusion can be drawn as follows:

- As the width of surface pit defect increases, the yield load of thin film decreases extremely slowly, until the size coefficient of width equals approximately one unit, at which point the yield load experiences an obvious drop. When the size coefficient of width reaches approximately two units, the yield load is almost the same as that of the nanoindentation on a stepped surface.
- As the height of surface pit defect increases, the yield load of thin film decreases extremely slowly, until the size coefficient of height equals approximately half unit, at which point the yield load experiences an obvious drop. When the size coefficient of height reaches one unit, the yield load is almost the same as that of the nanoindentation on a stepped surface.
- The height of surface pit defect has more influence than the width on the yield load of thin film, which suggests that the height of the pit is a leading factor on the influence of yield load. Such investigation results in this simulation may have important directive to the defects testing or material application.

Based on such a size effect of surface defect in nanohardness in the present paper, a further work of surface defect effect might be interesting and worth focusing on if the surface defect is not a cavity but another material, which is usually seen in alloy.
